# Quantitative synteny scoring improves homology inference and partitioning of gene families

**DOI:** 10.1186/1471-2105-14-S15-S12

**Published:** 2013-10-15

**Authors:** Raja Hashim Ali, Sayyed Auwn Muhammad, Mehmood Alam Khan, Lars Arvestad

**Affiliations:** 1 KTH Royal Institute of Technology, School of Computer Science and Communication, Department of Computational Biology, Stockholm, Sweden; 2 Science for Life Laboratory, Karolinska Institutet Science Park, Solna, Sweden; 3 Swedish e-Science Research Center, Sweden; 4 Department of Numerical Analysis and Computer Science, Stockholm University, SE-100 44, Stockholm, Sweden

## Abstract

**Background:**

Clustering sequences into families has long been an important step in characterization of genes and proteins. There are many algorithms developed for this purpose, most of which are based on either direct similarity between gene pairs or some sort of network structure, where weights on edges of constructed graphs are based on similarity. However, conserved synteny is an important signal that can help distinguish homology and it has not been utilized to its fullest potential.

**Results:**

Here, we present GenFamClust, a pipeline that combines the network properties of sequence similarity and synteny to assess homology relationship and merge known homologs into groups of gene families. GenFamClust identifies homologs in a more informed and accurate manner as compared to similarity based approaches. We tested our method against the Neighborhood Correlation method on two diverse datasets consisting of fully sequenced genomes of eukaryotes and synthetic data.

**Conclusions:**

The results obtained from both datasets confirm that synteny helps determine homology and GenFamClust improves on Neighborhood Correlation method. The accuracy as well as the definition of synteny scores is the most valuable contribution of GenFamClust.

## Background

Gene family classification is an important pre-requisite in Bioinformatics studies and enables, e.g., phylogenetic and structural analysis. Proteins translated from related genes (homologs) tend to have similar structure and function and most of their chemical properties are also similar [1]. One of the initial tasks in genome analysis, given a novel genome, is to find homology between genes and then to use this homology information to make a rough guess about the properties of each gene as well as to construct the phylogenetic tree from these gene families. Due to the importance of gene family classification, it has become one of the most active fields of research in Bioinformatics and bioinformaticians have employed different algorithms to detect homology and to partition detected homologs into gene families.

The pioneers of homology inference algorithms use similarity-based methods, typically employing BLAST [2,3] as a subroutine, like Reciprocal Bidirectional Hits (RBH) [4] and Clusters of Orthologous Groups (COGs) [5]. Other examples of similar algorithms are SiLiX [6] and BlastClust [7] that apply threshold on BLAST output, e.g., E-value and/or percentage identity, and perform single linkage clustering [8]. Despite speed and simplistic computations, they lack the sensitivity to infer homology for more divergent and highly evolving gene families, e.g., in the presence of differential gene loss and/or domain recombination events [9-11]. The next class of algorithms use sequence clustering techniques and examines a wide range of BLAST hits. Well-known examples are TribeMCL [12], OrthoMCL [13], InParanoid [14], and MultiParanoid [15], which are applicable on large datasets and are more accurate than simple BLAST based methods. The next generation of homology inference algorithms improved the accuracy and the time and/or memory complexity requirements and include algorithms like Neighborhood Correlation [16], HiFiX [17], PHYRN [18], COCO-CL [19] etc. and infer homologs by extracting evidence from network structure of BLAST hits or multiple sequence alignments.

The algorithms mentioned previously are all based on sequence similarity. Other algorithms have been designed that do not infer homology between genes but instead retrieve chromosomal regions that share homology. Given the chromosomal homology information, one can infer homologous genes by using similarity matches in the region. Examples are R-window [20] and max-gap [21], which use the concept of "gene teams" (conserved gene clusters) [22]. Popular software that implement these algorithms or variants thereof are SynBlast [23], MCScanX [24], Cyntenator [25] and DAGChainer [26]. However, homology inference from these software require further processing of results and homology is not a direct result from these algorithms and software.

At present, there is a relative lack of methods that assess homology by using synteny heuristics directly and not through implicit computation of syntenic regions. The few algorithms that use synteny directly for homology inference are not able to give an objective quantitative measure of synteny (capture synteny information in a score) for a given pair of gene. As an example, SYNERGY, a species-tree aware and synteny-based method, showed impressive results on yeast dataset [27]. However, the method is not general enough for use with all datasets [28]. An issue for using synteny information in this way is the fragmentation in genome assemblies, which may handicap current synteny based software. Alternative synteny-based strategies that may avoid this pitfall define synteny by using a fixed sized neighborhood (termed local synteny). Jun et al. [29] have used this definition to identify orthologs and have shown comparative results with other similarity-only based approaches. Another approach based on local synteny that also takes into account evidence from multiple genomes is SYNS (SYNtenic teamS) and has been shown to work on five Protoploid yeasts [30]. These and other such strategies generally define homology in the neighborhood by applying a threshold on the BLAST E-values, which has been shown by Joseph et al. [31] to be a weak indicator of homology.

We propose a novel gene similarity and synteny based pipeline that makes use of network structure for both similarity and synteny. First, it is a method based on evidence for conserved gene order across many genomes instead of only two genomes directly. Second, it is the first method to calculate synteny scores based on the Neighborhood Correlation score [31] (NC) instead of BLAST E-value and defines a quantitative synteny score. Third, there is a noticeable gain in accuracy when combining NC and synteny score compared to NC alone. Fourth, the pipeline is robust to fragmentation in genome assemblies and can reliably be employed to most data sets. GenFamClust is available as a single, user-friendly Java command line tool that provides homology inference pipeline and clustering algorithm implementations.

## Methods

Given a full list of sequences in Fasta format and information about order of each gene in a specified format, GenFamClust partitions the data into homologs and non-homologs by determining combined evaluated scores from NC [16,31] and synteny correlation (SyC) scores. From these classified homologs, GenFamClust constructs the gene families by using Single [8], Average [32] or Complete Linkage [33] clustering. GenFamClust searches for evidence of conserved synteny by computing the synteny correlation score for each pair of sequences that have acceptable sequence similarity. The main idea is that the advantages that NC has over BLAST based scores, can also be employed for synteny to make it more robust, standardized and accurate than the "gene teams" concept by making it based on evidence from multiple witnesses. While NC scores over 0.5 can in general be classified as homologs, GenFamClust uses synteny to assess homology for gene pairs with NC scores below 0.5.

### The data and pipeline

GenFamClust assumes that there are two sets of data; the query dataset *Q *and the reference dataset *R*. The query dataset *Q *consists of those genes for which homology relationships are inquired and classification into gene families is desired. The reference dataset *R *consists of those genes which will be used for finding evidence for conserved synteny but may not be of interest in the final analysis.

**Figure 1:**
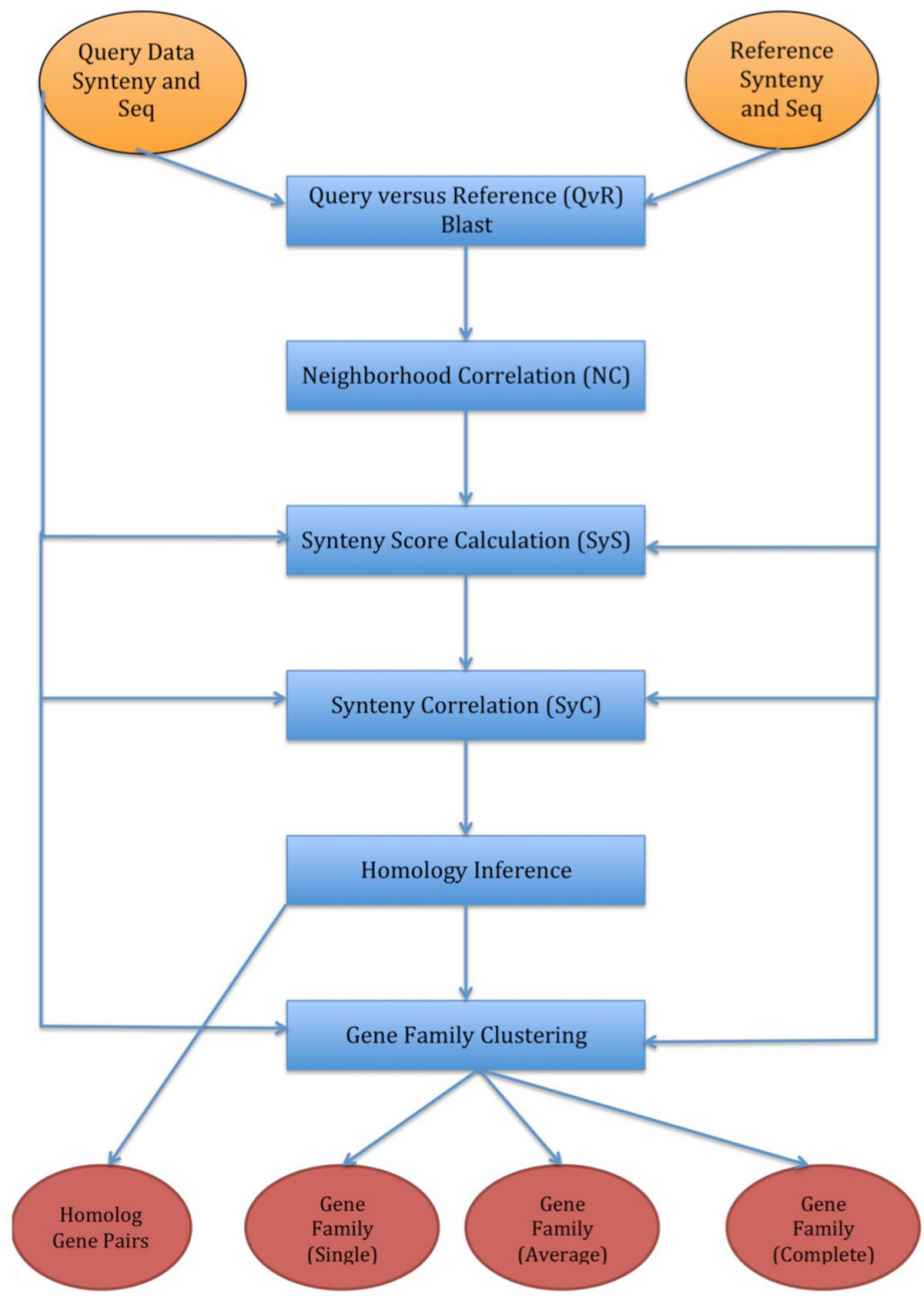
**General workflow of the GenFamClust pipeline**. Orange circles: the input to the pipeline; blue squares: module or process of the pipeline; red circles: the output of the pipeline. Arrows indicate data flow of the pipeline.

The input expected by the GenFamClust implementation is synteny files that contain information about the gene order and Fasta files containing protein sequences (exactly one per gene). Figure 1 describes the general workflow of the pipeline.

### Neighborhood Correlation calculation

We chose the Neighborhood Correlation score as given by Song et al. [16] as our measure of similarity. The attractive feature about this measure is that it is standardized, has a known range between 0 and 1, can easily be applied a threshold, has been shown to work well with diverse protein domain architectures and is more accurate than any simple BLAST based thresholds. We demand that NC score is above a threshold β and setting β = 0.3 ensures that most non-homologs are discarded while retaining virtually all homologs in the dataset. Furthermore, this limit helps reduce memory consumption. NC needs a lenient threshold on BLAST E-value [16]; For our experiments, we have chosen E = 0.1.

### Synteny score calculation

To compute SyC, we make use of a synteny score SyS(*g_1_, g_2_*) for two sequences *g_1 _*and *g_2_*. Let n(*g*) be the set of neighbor genes, upstream or downstream of *g*, at most at distance *k*, on a chromosome or contig. We define SyS(*g_1_,g_2_*) *= *max{NC(*a,b*) : *a *∈ n(*g_1_*), *b *∈ n(*g_2_*)}.

The purpose of *SyS *is to find evidence of homology of genes in n(*g_1_*) with genes in n(*g_2_*). SyS is only calculated for pairs (*g_1_, g_2_*) where NC(*g_1_, g_2_*) > β and at least one of *g_1 _*and *g_2 _*is in Q. Below β, NC is regarded sufficient to indicate that no homology exists. While the *QxQ *gene pairs indicate direct evidence for synteny in the query dataset, the *QxR *gene pairs provide indirect evidence within the reference dataset genes. Our experiments with the human-mouse dataset suggests setting *k *= 5 (see Additional File 1).

We tried four different functions to define a synteny score for a pair of genes and an assessment of the behavior of each method made us choose the "Maximum Score" method. See Additional File 1 for details on the alternatives and the assessment.

### Syntenic correlation calculation

For each gene pair (*g_1_, g_2_*) such that *g_1_, g_2 _***∈ ***Q *and NC(*g_1_, g_2_*) > β, GenFamClust computes synteny correlation scores, SyC, for using pairs with good NC score. Let $ncHits\left(g_{i}\right)=\left\{h|h\in Q\cup R,NC\left(g_{i},h\right)\geq \beta \right\}$and $H=ncHits\left(g_{i}\right)\cap ncHits\left(g_{j}\right)$, then

$$SyC\left(g_{i},g_{j}\right)=\frac{\underset{h\in H}{\sum }\left(SyS\left(g_{i},h\right)-\bar{SyS}\left(g_{i}\right)\right)\left(SyS\left(g_{j},h\right)-\bar{SyS}\left(g_{j}\right)\right)}{\sqrt{\underset{h\in H}{\sum }{\left(SyS\left(g_{i},h\right)-\bar{SyS}\left(g_{i}\right)\right)}^{2}}\sqrt{\underset{h\in H}{\sum }{\left(SyS\left(g_{i},h\right)-\bar{SyS}\left(g_{i}\right)\right)}^{2}}}$$

where $\bar{SyS}\left(g\right)$ is the average *SyS *taken over *H*.

Using SyC, we evaluate synteny as an evolutionary signal that can vary across lineages. Note that it is not necessary for *g_i _*and *g_2 _*to be found in synteny; 1) similarity to syntenic genes in reference species may support the homology of *g_i _*and *g_2 _*and 2) the range of SyC is 0-1 like NC.

### A combined score

NC(*g_1_, g_2_*) and SyC(*g_1_, g_2_*) scores are transformed into a single "strength of prediction" score using an elliptical function that evaluates the homology relationship between two genes. This strength of prediction variable has a range between 0 and 1 and increases consistently as NC and/or SyC values increase. It is standardized, normalized and gives strength of prediction score for all homolog gene pairs. From rigorous testing on a human mouse dataset at different NC and SyC thresholds (described in Additional File 1), the best curve that has maximum individual family specificity and sensitivity is an ellipse that cuts SyC at around 1.0 and NC at around 0.5. For a gene pair (*g_1_, g_2_*), the formula for calculating the evaluation value h(*g_1_, g_2_*) is given by

h(g_1_, g_2_) = NC(g_1_, g_2_)^2 ^+ 0.25 * SyC(g_1_, g_2_)^2 ^- 0.25.

### Gene family clustering

Depending on the requirement of type of gene families required, we have tested three standard algorithms. GenFamClust has custom implementations of single linkage, complete linkage and average linkage clustering, which are tailored for using transformed scores, are memory efficient and thus suitable for even very large datasets. For single linkage and complete linkage, gene pairs (*g_1_, g_2_*) with h(*g_1_, g_2_*) > 0 were considered. For average linkage clustering, the average similarity threshold score 0.25 (described in Additional File 1) has been set.

## Results

### Validation on a simulated dataset

To enable validation on data that we fully understand, we generated data using ALF [34], which is a software that simulates major evolutionary forces for genome rearrangement. The details of parameter settings used for generating this dataset are given in Additional File 1.

We selected Mus musculus chromosome 18 as input to ALF due to its nominal size of 497 genes. We then performed six simulations by varying translocation rate, values 0.0002, 0.0025 and 0.005, and substitution rate, from 100 to 250 PAM, to test GenFamClust for varying levels of gene order and gene content conservation. We used default parameters setting for all other options and turned off parameters related to Gene Inversion, Lateral Gene Transfer (LGT), Fission, Fusion and Pseudogenization events without loss of generality. For this dataset, since no referenced data R has been defined, Query data Q also acts as the reference data.

Table 1 illustrates the comparison between NC and GenFamClust for the simulated dataset, where each cell represents the absolute difference in number of true gene families and inferred by using a clustering algorithm on scores from NC and the combined score (NC and SyC). Clearly, GenFamClust outperforms NC in determining the gene families, where the resulting number of gene families formed by GenFamClust is closer to actual gene families in almost all cases. This indicates that SyC is informative and improves on NC scores alone. Datasets 1, 2 and 3, which have higher synteny conservation, are better approximated by both methods, which emphasizes the dependence of NC and GenFamClust on gene content conservation.

**Table 1:** Absolute difference between number of gene families determined by NC and those determined by GenFamClust.

		Dataset					
	**Transl. rate**	.0002	.0025	.005	.0002	.0025	.005

	**Dupl. rate**	.0085	.0085	. 0085	.006	.006	.006

	**Subst. rate**	100	100	100	250	250	250

**Clustering**	**Algorithm**	**1**	**2**	**3**	**4**	**5**	**6**

Average Linkage	NC	83	10	39	1211	552	493

	GenFamClust	32	7	23	751	457	437

Complete Linkage	NC	127	35	68	1316	608	560

	GenFamClust	58	16	53	821	503	489

Single Linkage	NC	59	6	21	1115	502	440

	GenFamClust	6	16	1	631	397	379

Extant gene families	-	329	289	382	241	258	233


Each cell represents the absolute difference between number of extant gene families (observable at leaves) (last row) and the number of gene families determined by the corresponding gene family algorithm for the corresponding dataset with the corresponding linkage algorithm. For NC, the threshold of 0.5 was used while for GenFamClust, the elliptical curve with NC = 0.5 and SyC = 1.0 was used.

### Human versus mouse dataset

The Human-Mouse dataset is from Ensembl Genes 69 [35], has human and mouse genomes as query, and has a reference dataset consisting of complete genomes from eighteen eukaryotic species, ranging from yeast to mammals(including human and mouse). A gold standard dataset was available in the form of twenty homologous gene families of human and mouse identified by Song et al. [16].

Since GenFamClust requires whole genome information, we used the human and mouse genome data, extracted from Ensembl, as our query sequences. For reference sequences, we selected genomes evenly distributed over the Species tree of life provided by Ensembl [36].

Song et al. suggested 20 gene families in human and mouse based on literature in their paper [16]. These families are diverse and contain single as well as multi-domain families; contain very small families to very large families; and vary from very conserved families to highly divergent sequence families (shown in Additional File 1). With this known excellent gold standard, it was very logical to test our approach on this dataset and compare with similarity only software.

#### Validating GenFamClust

**Figure 2:**
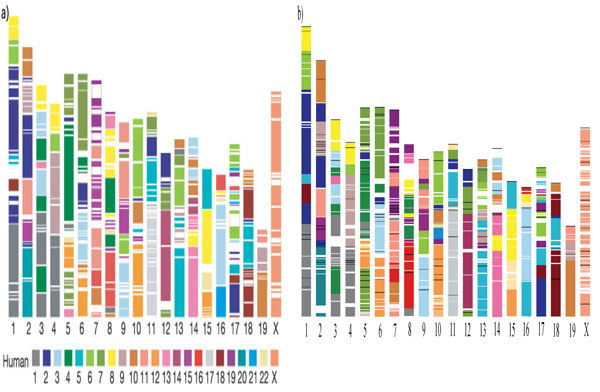
**Mapping of human genome onto mouse chromosome using synteny computed a) from BLAST hits and b) from SyC**. A synteny image of the mouse genome, as compared to human genome using a) BLAST scores and dotplots in the original human sequencing paper [33] (reproduced with permission from Nature Publishing Group) and b) using NC and SyC scores between human and mouse from GenFamClust. a) has been computed by using synteny information from the dot plot for whole genomic matching regions of 300 kbp size or more, while b) has been computed by determining gene teams of at least size 5 and with a minimum NC and SyC of 0.5. Each chromosome in b) has been normalized by the size of chromosome for comparison with a). White lines represent lack of synteny in a) and b), while black lines (only in b)) represent break in synteny in neighboring gene within the same chromosome. Counting all breaks in syntenic regions (white lines, black lines and change of chromosome), there are 294 syntenic segments with 208 regions (change of chromosome only) for b) as compared to 342 syntenic segments with 217 blocks of consistent color.

GenFamClust was applied to the human and mouse dataset and was checked for the results on the gold standard data of twenty families. The first paper published after sequencing of mouse genome gave a synteny-based match of mouse genome with the human genome [37]. Such a large number of conserved syntenic regions and the level of conservation provides a strong argument in favor of using synteny to support gene homology inference. To validate that the synteny score of GenFamClust is capturing gene order conservation information, we applied GenFamClust on the human and mouse datasets and found that GenFamClust could replicate the original image [37] almost perfectly: 342 syntenic segments with 217 blocks of consistent color in the original image vs 294 syntenic segments with 208 blocks of consistent color using NC and SyC). The few regions and segments missed by our approach did not contain genes or contained less than five genes. Figure 2 is a comparison between the original image and our results.

#### Comparison with Neighborhood Correlation without synteny

**Figure 3:**
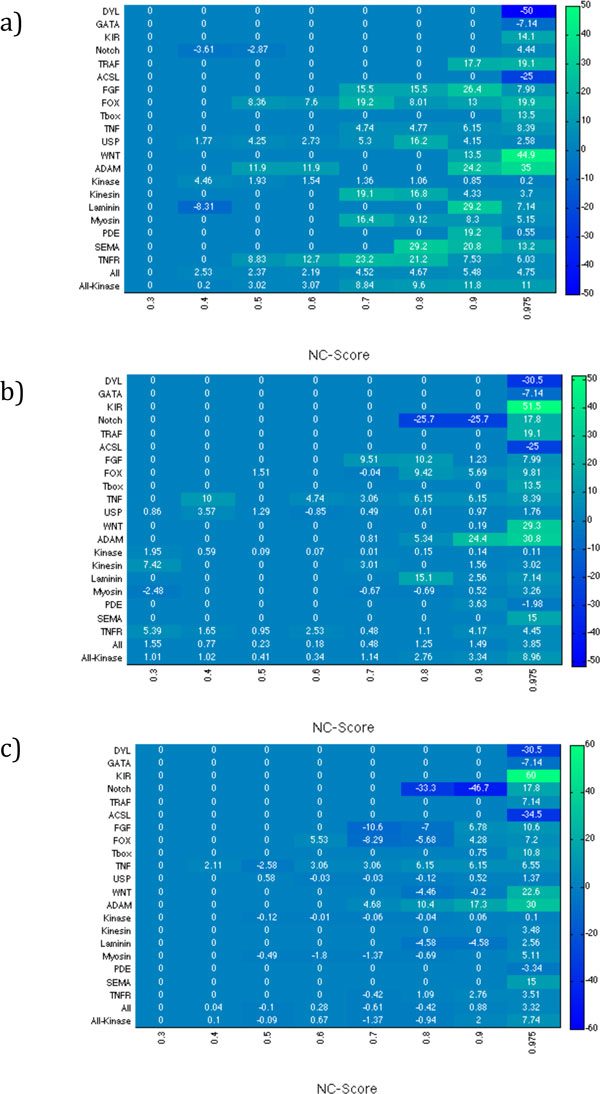
**Evaluation of clustering on transformed scores at various NC scores with SyC cut at 1.0 versus NC scores alone**. Figure enumerates and displays the comparison of gene families formed by a) Single Linkage Clustering, b) Average Linkage and c) Complete Linkage. The value in each cell represents the difference between quality scores of clusters generated by GenFamClust and quality scores of clusters generated by NC alone for corresponding cell on human mouse test dataset. Green cells represent the families where GenFamClust outperforms the NC method, dark blue cells represent the families where NC outscores GenFamClust, and blue cells represent the families where both quality scores are equal. The intensity of green and blue indicates the difference in percentage between the two approaches, where darker color shows greater difference.

We applied GenFamClust and NC to complex and diverse cases of the gold standard dataset from Song et. al [16]. We compared the performance of Neighborhood Correlation software to the performance of GenFamClust according to *F(i, j)*, the harmonic mean of precision (*P(i, j) *= fraction of elements in cluster *j *that are members of family *i*) and recall (*R(i, j) *= fraction of members of family *i *that are found in cluster *j*) [31]. *F(i,j) *(shown in Figure 3) is determined by following formula.

$$f\left(i,j\right)=\frac{2P\left(i,j\right)R\left(i,j\right)}{P\left(i,j\right)+R\left(i,j\right)}$$

The results, shown in Figure 3, clearly demonstrate that we have a marked improvement in terms of accuracy for single linkage (on average 3.81 percentage points) and average linkage clustering (on average 1.63 percentage points) while we have maintained the accuracy shown by the Neighborhood Correlation alone in the complete linkage-clustering (on average 0.44 percentage points) algorithm. In particular comparing the two quality scores at the proposed threshold of 0.5 for NC, GenFamClust outperforms NC in single linkage (2.37 percentage points) and average linkage (0.23 percentage points) clustering while has a minute difference (-0.1 percentage points) with NC in complete linkage clustering.

We also examined the effect of varying NC values while SyC score remains constant at 1.0 and vice versa. Lowering NC threshold improves the overall and all-kinase quality scores. However, it can be observed that the small sized families tend to suffer with low NC values. Therefore, it is logical to choose a NC value threshold that is best able to define individual families for all three clustering algorithms. In this regard, a NC value threshold of just around 0.5 seems to be the most appropriate (complete tables in Additional File 1). Joseph et al. made the same deduction in the follow up paper of Neighborhood Correlation as well [31]. Similarly, for GenFamClust and NC value 0.5, an evaluation curve cutting SyC axis at 1.0 on SyC seems to provide the best results (Data and tables in Additional File 1).

## Discussion

Conserved gene order is one of the properties that can aid in identifying homologs along with similarity. In this paper, we combined gene order and content conservation to infer homology. We use the concept of local synteny as well as gain evidence from multiple genomes, similar to [29,30]. However, we suggest a way to quantify synteny and combine it with similarity information before doing the actual classification. Moreover, we avoid the pitfalls of BLAST scores by building on NC [31].

### Syntenic orthologs versus non-syntenic Orthologs

Since orthology is generally extracted from direct similarity measures, orthologs with syntenic support have an extra degree of confidence in their prediction. Depending on the requirements for determining gene families, if split families is not problematic but accurate clustering is a requirement, then syntenic orthologs can act as a good dataset. Furthermore, as displayed by Wolf et al. [38], syntenic orthologs can act as validation data for confirming the results from different techniques.

### Choice of reference dataset

The choice of reference dataset is highly important as it has profound impact on the Neighborhood Correlation scores for both similarity and synteny. The reference data must reflect the similarity and synteny information for the query dataset accurately. While there is no upper bound on the amount of reference data, there are practical limitations as well as usability issues for the size of the reference data set; having many species with little divergence times will have redundant similarity and synteny information, which only adds to the computational burden without adding any new information. On the other side of spectrum, if no reference data is available, the query data itself serves as reference data. In general, reference data should be able to capture the synteny and similarity relationships for the query data e.g. by choosing a few representative species from each branch of a known species tree from which query dataset is taken from.

### Advantages of using Query versus Reference Blast

All similarity-based programs mentioned in this study require All-versus-All Blast results for gene family classification. GenFamClust takes advantage of network structure employed in NC for similarity and performs a Query versus Reference Blast only. Then, the Reference versus Reference Blast results are appended to these results and passed onto the next module for NC calculation. As the size of R is fixed, the size of Q varies and is the determining factor of the time taken by the Blast module. While for an All-versus-All Blast, it would take *O((n+m)^2^) *time, this version of Blast takes *O(mn+n^2^) *time, where *m *is the size of *Q *and *n *is the size of *R*. This comparison is, of course, only meaningful when *m*>>*n*. Furthermore, the Blast results for Reference versus Reference dataset can be reused giving the effective time complexity of *O(nm)*.

## Conclusions

Clustering sequences into meaningful families and to infer the true evolutionary history of widely diverse set of genes are difficult tasks. While the clustering techniques are relatively long known and mostly standard, homology inference is the defining step for determining accurate gene families. However, homology inference is an Achilles heel of determining reliable gene families. Methodologies only based on similarity have long been proposed for homology inference without taking account of synteny. However, a sensible combination of sequence similarity and synteny would perform better than only similarity-based approaches. In this work, we have proposed GenFamClust, a novel pipeline that is first to make use of network structure of synteny across multiple genomes. It provides an objective way of assessing synteny for a gene pair as well as a noticeable improvement in accuracy as compared to a similarity-only algorithm. We suggest that GenFamClust is a good framework due to its ability to handle larger genomes, large and diverse datasets spread across a variety of species from Eukaryotes, as well as across varying protein domain architectures from single domain to conserved and varying multi-domain proteins. Another feature of GenFamClust is its ability to work and define synteny with fragmented gene assemblies. Moreover, the Java implementation of GenFamClust is user friendly and easy to deploy and use by the general community.

## Competing interests

The authors declare that they have no competing interests.

## Authors' contributions

RHA designed and implemented the algorithm for GenFamClust, prepared the multi-species datasets, performed comparative analysis with NC and drafted the manuscript. SAM collaborated in designing the algorithm, performed statistical analysis of all datasets and prepared the simulated dataset. MAK participated in the design of the study and aided in evaluation of human-mouse dataset. LA conceived of the study, participated in its design and coordination and helped to draft its manuscript.

## Supplementary Material

Additional file 1 **Supplementary materials**. Data descriptions, technical details, and additional results. Click here for file

## Declarations

RHA was funded by Higher Education Commission of Pakistan (HEC). SAM was funded by EuroSPIN (an Erasmus Mundus joint doctoral program) and Swedish e-Science Research Center (SeRC). MAK was funded by UET Peshawar. Publication charges for this article were paid by the KTH Royal Institute of Technology.

This article has been published as part of BMC Bioinformatics Volume 14 Supplement 15, 2013: Proceedings from the Eleventh Annual Research in Computational Molecular Biology (RECOMB) Satellite Workshop on Comparative Genomics. The full contents of the supplement are available online at http://www.biomedcentral.com/bmcbioinformatics/supplements/14/S15.
