# Supplementary material for: Quantitative synteny scoring improves homology inference and partitioning of gene families
Source: BMC Bioinformatics. 2013 Oct 15;14(Suppl 15):S12. doi: 10.1186/1471-2105-14-S15-S12 (PMC3852004; doi:10.1186/1471-2105-14-S15-S12)
Supplement: Additional file 1 — Supplementary materials. Data descriptions, technical details, and additional results. [file 1471-2105-14-S15-S12-S1.PDF]

Quantitative synteny scoring improves homology inference and partitioning of gene families

Supplementary Data

Ali RH, Muhammad SA, Khan MA and Arvestad L

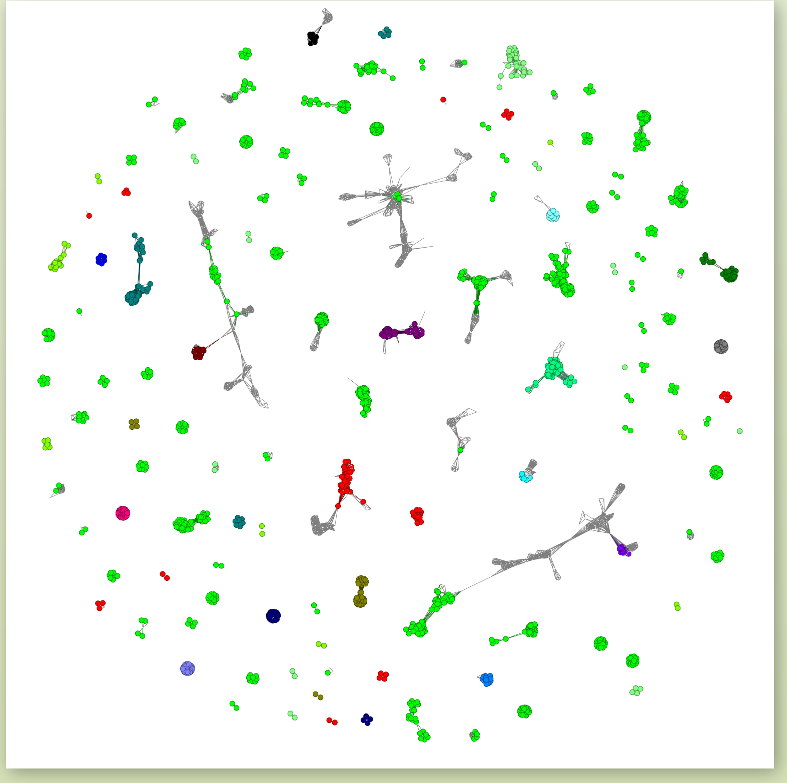

Table of contents

TABLE OF CONTENTS 2

1) Evaluating Synteny Scores method and optimal neighborhood size 2
Abbreviations: 2
Heuristic: 2
Mathematical formulas of all functions: 2
Results: 3
Deduction: 3

2) Values of major and minor axis cut to choose for best elliptical curve 4
Results: 5
Deduction: 6

3) Selection of thresholds for clustering algorithms 6
Single and complete linkage algorithms 6
Average linkage algorithm 6

4) Characterization of datasets 7
Simulated Dataset 7
Human versus Mouse dataset 7

5) Accuracy of QvR Blast vs All-versus-All-Blast: 8

6) Interesting notes: 9
i) Why NC not BLAST for defining similarity 9
ii) Advantages of using NC scores for calculating synteny scores 9

1) Evaluating Synteny Scores method and optimal neighborhood size

Abbreviations:

MS = Max Score
ASA = All Sum Analysis
SBGANNA = Sum Best Gene And Nc-hit Neighbor Analysis
SBGNA = Sum Best Gene Neighbor analysis

Dataset: Human versus Mouse dataset.
# of Query species = 2 (human and mouse)
# of genes in query species = 44736 genes
# of NC-hits between query genes > 0.3 = 1,435,696 hits
# of NC-hits > 0.5 = 918,780 hits (about 64% of all NC hits > 0.3)
# of NC-hits < 0.5 = 516,916 hits (about 36% of all NC-hits > 0.3)

Heuristic:

i) NC(g_1, g_2) > 0.5 means that most likely, g_1 and g_2 are homologs (Joseph et. al [31]) and ii) NC(g_1, g_2) < 0.5 means that most likely, g_1 and g_2 are not homologs (Joseph et. al [31]). Assuming that SyC(g_i,g_j) > 0.5 also indicates homology, the method and the k that maximizes i) maximizes the number of gene pairs g_i and g_j with SyC(g_i, g_j) > 0.5 for NC-hits(gi, gj) > 0.5 and ii) maximizes the number of gene pairs g_i and g_j with SyC(g_i, g_j) < 0.5 for NC-hits(g_i, g_j) < 0.5.

Mathematical formulas of all functions:

For each gene $g_i \in Q$, and for each gene pair (g_i, g_j) such that NC(g_i, g_j) > 0.3, do the following.

- **Maximum Score Analysis:** When assessing the synteny score of g_i and g_j, return the maximum NC-score among the genomic neighbors of g_i and g_j.

$$SyS(g_i,g_j) = argmax\{NC(a,b) : a \in neighbor(g_i) \ \& \ b \in neighbor(g_j)\}$$

- **Sum Up Every Hit Analysis:** Accumulates all the available NC-hit scores in the neighborhood of g_i and g_j.

$$SyS(g_i,g_j) = \sum_{a \in neighbor(gi) \ \& \ b \in neighbor(gj)} NC(a,b)$$

- **Sum Best Gene Neighborhood Analysis:** Finds the hit with maximum score for each of the $2k$ neighbors of g_i with the neighbors of g_j and sums them up.

$$h = argmax_{b \in neighbor(gj)} \{NC(a,b) : a \in neighbor(gi)\}$$
$$SyS(g_i,g_j) = \sum_{a \in neighbor(gi)} NC(a,h)$$

- **Sum Best Gene and NC Neighborhood Analysis:** Finds the best hit with maximum score for each of the $2k$ neighbors of g_i with the neighbors of g_j as well as for the $2k$ neighbors of g_j with the neighbors of g_i and sums these $4k$ values.

$$h_a = argmax_{b \in neighbor(gj)} \{NC(a,b) : a \in neighbor(g_i)\}$$
$$h_b = argmax_{a \in neighbor(gi)} \{NC(a,b) : b \in neighbor(g_j)\}$$
$$SyS(g_i,g_j) = \sum_{a \in neighbor(gi)} NC(a,h_a) + \sum_{b \in neighbor(gj)} NC(h_b,b)$$

Results:

i) For Heuristic i), where each cell represents percentage of SyC(g_i, g_j) > 0.5 for NC-hits(g_i, g_j) > 0.5 (probably homologs).

NC (g_i, g_j) > 0.5 918,780

Method	Neighborhood Size						
	1	2	3	4	5	6	7
ASA	39.89%	37.99%	35.56%	32.78%	28.92%	27.29%	27.02%
MS	48.03%	51.21%	55.66%	56.75%	57.54%	57.62%	57.02%
SBGANNA	39.78%	37.68%	34.87%	31.56%	28.34%	26.97%	26.92%
SBGNA	39.72%	37.50%	34.22%	30.45%	27.91%	26.83%	26.77%

Table 1 - Each cell represents percentage of SyC(g_i, g_j) > 0.5 for NC-hits(g_i, g_j) > 0.5

ii) For Heuristic ii), where each cell represents percentage of SyC(g_i, g_j) < 0.5 for NC-hits(g_i, g_j) < 0.5 (probably non-homologs).

NC (g_i, g_j) > 0.5 516,916

Method	Neighborhood Size						
	1	2	3	4	5	6	7
ASA	80.43%	80.89%	81.67%	82.04%	82.98%	83.56%	83.78%
MS	78.26%	74.48%	71.76%	69.49%	69.14%	69.02%	69.14%
SBGANNA	80.59%	81.21%	81.92%	82.78%	83.26%	83.68%	83.85%
SBGNA	80.67%	81.32%	81.97%	82.84%	83.29%	83.71%	83.88%

Table 2 - Each cell represents percentage of SyC(g_i, g_j) < 0.5 for NC-hits(g_i, g_j) < 0.5

Deduction:

Keeping in mind the heuristic defined earlier, Maximum Score method outperforms all other methods considerably in the first heuristic while all other methods seem to do equally well in the second heuristic than Maximum Score. However, the

behavior of Maximum Score is consistent throughout both tables and keeps on increasing/decreasing till k = 5 after which the effect of noise begins to be visible (k = 6, 7). Therefore, if maximum score method is chosen, then k = 5 will be the best setting for neighborhood size. On the other hand, the other methods behave differently for different values of k in particular for heuristic 1. The following figure depicts the general properties of each method. Therefore it would be safe to deduce that the ideal setting for calculating synteny score would be with maximum score method and with neighborhood size k = 5.

Our choice differs from the one chosen by Jun et al. [29], where they chose number of neighboring genes as 3 because Jun et al. have utilized more than one hit. As shown below, if more than one hit is to be utilized, then k = 3 has the best result as that is the maximum value for which bi-modal distribution exists (also deduced by Jun et al.). However, defining range on BLAST hits for homologs is difficult and the more the hits in neighborhood, the better it will be. But NC is ranged between 0 and 1 and therefore choosing the maximum is more meaningful in our case as compared to Jun et al.

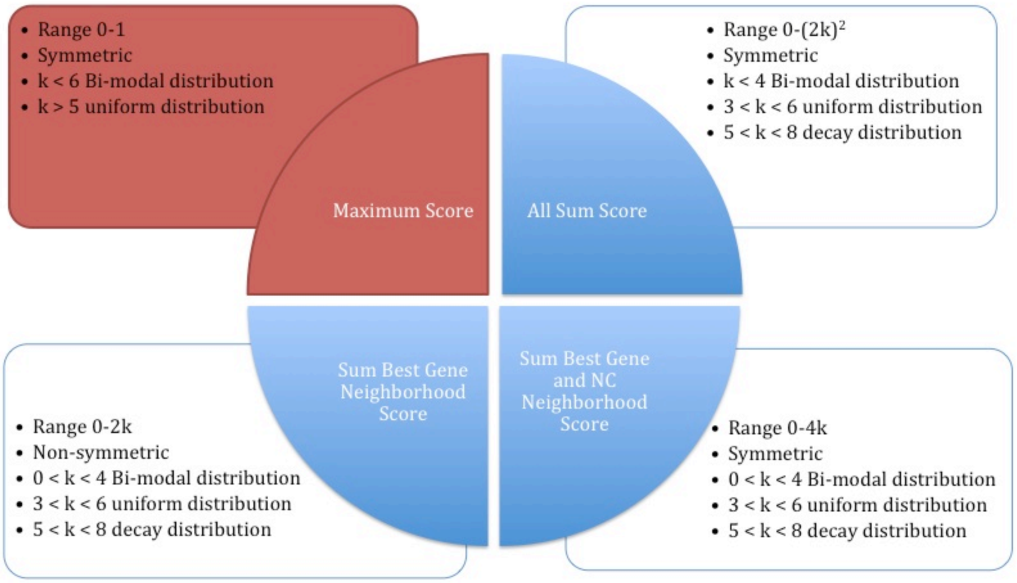

Figure 1 – Properties of different methods for calculating synteny scores**.**

2) Values of major and minor axis cut to choose for best elliptical curve

Dataset: Human versus mouse dataset.

Method: Use a selected set of evaluation functions that cover most of the elliptical curves and evaluate the clustering quality for each curve for each clustering algorithm using the test dataset (1558 proteins with 20 gene families). So, we selected SyC = 0 to SyC = 1 with an increment of 0.1 with NC = 0.5 (taken from Joseph et al [31]) for the first set of runs. Range of NC is 0.3-1 and SyC is 0-1. Here SyC values indicate the value of SyC at NC=0.3 and not the minor axis cut (Do not confuse SyC value as the minor axis cut value). E.g. if the points (SyC = 0.8, NC = 0.3 and SyC = x, NC=0.5) is on the elliptical boundary, x can be calculated using the standard ellipse formula (x = 1).

Criteria: The evaluation curve that best explains 1) Overall 2) All-kinase and 3) smaller gene families because smaller families contribute less to the overall and all-kinase scores but they are equally important for all families.

Results:

	SNC cut = 0.1	0.2	0.3	0.4	0.5	0.6	0.7	0.8	0.9	1	NC Thr
DVL	1	1	1	1	1	1	1	1	1	1	1
GATA	1	1	1	1	1	1	1	1	1	1	1
KIR	1	1	1	1	1	1	1	1	1	1	1
Notch	0.182	0.182	0.182	0.182	0.182	0.182	0.182	0.182	0.182	0.182	0.211
TRAF	1	1	1	1	1	1	1	1	1	1	1
ACSL	1	1	1	1	1	1	1	1	1	1	1
FGF	1	1	1	1	1	1	1	1	1	1	1
FOX	0.868	0.868	0.868	0.868	0.868	0.868	0.868	0.868	0.784	0.784	0.784
Tbox	1	1	1	1	1	1	1	1	1	1	1
TNF	0.401	0.401	0.401	0.401	0.401	0.401	0.401	0.401	0.401	0.401	0.401
USP	0.714	0.714	0.714	0.634	0.634	0.461	0.461	0.447	0.418	0.418	0.404
WNT	1	1	1	1	1	1	1	1	1	1	1
ADAM	1	1	1	1	1	1	1	1	1	1	0.881
Kinase	0.172	0.137	0.136	0.128	0.128	0.110	0.104	0.089	0.087	0.082	0.070
Kinesin	1	1	1	1	1	1	1	1	1	1	1
Laminin	0.386	0.386	0.386	0.386	0.386	0.386	0.710	0.710	0.710	0.710	0.710
Myosin	1	1	1	1	1	1	1	1	1	1	1
PDE	0.820	0.820	0.820	0.820	0.820	0.820	0.820	0.820	0.820	0.820	0.820
SEMA	1	1	1	1	1	1	1	1	1	1	1
TNFR	0.612	0.612	0.612	0.612	0.612	0.612	0.612	0.612	0.612	0.612	0.524
All	0.458	0.437	0.437	0.429	0.428	0.409	0.411	0.401	0.394	0.391	0.377
# of clusters	93	95	96	99	100	104	106	110	114	116	124
All-Kinase	0.879	0.879	0.879	0.869	0.869	0.849	0.849	0.848	0.835	0.835	0.818

Table 3.1 - Results obtained for an elliptical function with NC= 0.5 and varying SYC values between 0 and 1 at NC=0.3. Single Linkage Clustering algorithm has been used to form clusters. Last column is the clustering quality result by thresholding on NC alone. Orange indicates maximum clustering quality value 1 by NC. Red cells indicate poorer, green indicates better and white indicates equally good clustering quality by the evaluation function for the family at the specified SyC value.

	SNC cut = 0.1	0.2	0.3	0.4	0.5	0.6	0.7	0.8	0.9	1	NC Thr
DVL	1	1	1	1	1	1	1	1	1	1	1
GATA	1	1	1	1	1	1	1	1	1	1	1
KIR	1	1	1	1	1	1	1	1	1	1	1
Notch	1	1	1	1	1	1	1	1	1	1	1
TRAF	1	1	1	1	1	1	1	1	1	1	1
ACSL	1	1	1	1	1	1	1	1	1	1	1
FGF	0.833	0.833	0.833	0.833	0.833	0.833	0.833	0.833	0.833	0.833	0.833
FOX	0.508	0.513	0.513	0.489	0.489	0.489	0.489	0.489	0.489	0.489	0.474
Tbox	1	1	1	1	1	1	1	1	1	1	1
TNF	0.301	0.301	0.301	0.301	0.301	0.301	0.301	0.301	0.301	0.301	0.301
USP	0.249	0.271	0.260	0.251	0.255	0.248	0.248	0.248	0.248	0.248	0.235
WNT	1	1	1	1	1	1	1	1	1	1	1
ADAM	0.833	0.833	0.881	0.881	0.881	0.881	0.881	0.881	0.881	0.881	0.881
Kinase	0.043	0.043	0.043	0.043	0.042	0.042	0.042	0.042	0.042	0.042	0.041
Kinesin	0.537	0.578	0.774	0.563	0.563	0.563	0.563	0.563	0.563	0.563	0.563
Laminin	1	1	1	1	1	1	1	1	1	1	1
Myosin	0.673	0.679	0.679	0.692	0.692	0.692	0.692	0.692	0.692	0.692	0.692
PDE	0.586	0.586	0.566	0.566	0.566	0.566	0.566	0.566	0.566	0.566	0.566
SEMA	1	1	1	1	1	1	1	1	1	1	1
TNFR	0.206	0.236	0.264	0.264	0.264	0.264	0.264	0.257	0.257	0.257	0.248
All	0.297	0.301	0.309	0.300	0.300	0.300	0.300	0.299	0.299	0.299	0.297
# of clusters	174	171	171	172	170	171	171	173	173	173	179
All-Kinase	0.644	0.653	0.673	0.652	0.653	0.652	0.652	0.651	0.651	0.651	0.647

Table 3.2 – Same as Table 3.1 but with Average Linkage Clustering instead of Single Linkage Clustering.

	SNC cut = 0.1	0.2	0.3	0.4	0.5	0.6	0.7	0.8	0.9	1	NC Thr
DVL	1	1	1	1	1	1	1	1	1	1	1
GATA	1	1	1	1	1	1	1	1	1	1	1
KIR	1	1	1	1	1	1	1	1	1	1	1
Notch	1	1	1	1	1	1	1	1	1	1	1
TRAF	1	1	1	1	1	1	1	1	1	1	1
ACSL	1	1	1	1	1	1	1	1	1	1	1
FGF	0.589	0.589	0.589	0.589	0.589	0.589	0.678	0.678	0.678	0.678	0.678
FOX	0.347	0.381	0.434	0.440	0.469	0.469	0.472	0.472	0.472	0.472	0.472
Tbox	1	1	1	1	1	1	1	1	1	1	1
TNF	0.228	0.228	0.228	0.228	0.228	0.228	0.228	0.228	0.228	0.228	0.254
USP	0.103	0.103	0.116	0.130	0.145	0.145	0.145	0.149	0.147	0.147	0.143
WNT	1	1	1	1	1	1	1	1	1	1	1
ADAM	0.820	0.881	0.881	0.881	0.881	0.881	0.881	0.881	0.881	0.881	0.881
Kinase	0.027	0.028	0.030	0.030	0.032	0.032	0.032	0.032	0.032	0.033	0.033
Kinesin	0.253	0.261	0.261	0.312	0.312	0.312	0.312	0.312	0.312	0.312	0.312
Laminin	0.792	0.669	0.669	0.729	0.729	0.729	0.729	0.729	0.729	0.729	0.729
Myosin	0.338	0.338	0.361	0.458	0.458	0.463	0.476	0.476	0.476	0.476	0.481
PDE	0.493	0.566	0.566	0.566	0.566	0.566	0.566	0.566	0.566	0.566	0.566
SEMA	1	1	1	1	1	1	1	1	1	1	1
TNFR	0.156	0.137	0.137	0.137	0.136	0.147	0.147	0.147	0.147	0.147	0.147
All	0.236	0.241	0.245	0.252	0.255	0.256	0.259	0.259	0.259	0.260	0.260
# of clusters	250	239	232	221	214	211	210	210	211	211	211
All-Kinase	0.523	0.531	0.540	0.556	0.561	0.562	0.569	0.570	0.569	0.569	0.570

Table 3.3 – Same as Table 3.1 but with Complete Linkage Clustering algorithm instead of Single Linkage.

Deduction:

We experimented and found that NC = 0.5 gave the best match of completeness and accuracy as well like Joseph et al.[31](data not shown). Then we look at the above tables and it can be argued that SyC=0.8 is possibly the best threshold at which individual families are generally performing well as well the overall clustering quality is also maintained. Keeping both these points in mind, we selected SyC = 0.8 as at this value, all three clustering methods perform well. Before or after this point, at least one family or overall clustering quality gets reduced in at least one clustering method.
Conclusions: NC=0.5 and SyC=0.8 (a=0.5 and b=1.0) provides the most sensitivity and specificity (in short clustering quality) for the human versus mouse dataset.

3) Selection of thresholds for clustering algorithms

Single and complete linkage algorithms

We selected similarity thresholds of 0.000001, which is approximately equal to 0 but not zero because we need all the homology pairs to compute single and complete linkage clusters. Since the range of evaluated homology scores is between 0 and 1, therefore setting a similarity threshold of almost 0 will capture all the pairs.

Average linkage algorithm

We selected 0.25 threshold on similarity for average linkage because close to 0, the behavior of average linkage is same as single linkage (due to the arithmetic rule that weighted averaging of a positive number and zero will always be greater than zero). On the other hand, since most of the dataset did not have synteny support, the homology values of most pairs were within 0-0.5 scores causing poor results on 0.5 threshold on similarity. Therefore, we chose the average of 0.5 and 0 and 0.25 gave a nice balance between single and complete linkage clusterings.

4) Characterization of datasets

Simulated Dataset

Following are the major parameter settings for the simulated dataset.

Simulation #	Translocation rate	Duplication rate	indel rate	indel size	Substitution rate	Number of genes
1	0.0002	0.0085	0.00005	25	100	4272
2	0.0025	0.0085	0.00005	25	100	3837
3	0.005	0.0085	0.00005	25	100	4065
4	0.0002	0.006	0.00005	25	250	4433
5	0.0025	0.006	0.00005	25	250	3940
6	0.005	0.006	0.00005	25	250	3899

Table 4 - Parameter settings used with ALF. Translocation rate was varied to check the effect of gene order conservation while changing the Substitution rate parameter varied gene content conservation. The Duplication rate parameter was changed to maintain the size of each gene tree, which is the number of genes in the whole dataset. Loss rate is set at 0.0005 for all simulations. The last column here is directly proportional to the number of genes present in the ancestral chromosome, which is also related to the extant gene families row in Main manuscript Table 1.

Human versus Mouse dataset

Selecting genomes and sequences for *R* in the Human-Mouse dataset

For reference sequences, we selected genomes evenly distributed over the Species tree of life provided by Ensembl [36]. The species selection was made on the basis that model organisms were preferred, and a genome should have a better assembly than other choices in its clade. When more than one model species with a well-assembled genome was found in a clade, they were all selected. When a clade had no well-assembled genome, any model species was considered, even with fragmented genome assembly. Since synteny is a gene-dependent characteristic, in case of many isoforms of the same gene (in particular in case of human and mouse), we selected the isoform with the longest sequence as the representative protein for each gene.

Gene Family Names	Members	Domain Architecture	Level of Conservation
DVL	6	Cons MultiDomain	High
GATA	12	Cons MultiDomain	High
KIR	9	Cons MultiDomain	High
Notch	8	Cons MultiDomain	High
TRAF	12	Cons MultiDomain	High
ACSL	10	Single	High
FGF	44	Single	Average
FOX	78	Single	Average
Tbox	30	Single	Average
TNF	31	Single	Average
USP	76	Single	Low
WNT	38	Single	Low
ADAM	44	Variable MultiDomain	Average
Kinase	900	Variable MultiDomain	Low
Kinesin	56	Variable MultiDomain	Average
Laminin	22	Variable MultiDomain	Average
Myosin	45	Variable MultiDomain	Average
PDE	44	Variable MultiDomain	Average
SEMA	38	Variable MultiDomain	Average
TNFR	55	Variable MultiDomain	Average
All	1558	Diverse	Diverse
All-Kinase	658	Diverse	Diverse

Table 5 - Table showing gene family names, members and domain architecture of each family of test dataset. The families capture diversity in the size, domain architecture and level of sequence conservation within each family. The largest and most complex gene family, by far, is Kinases and to avoid biases in quality score, All-Kinase consists of all proteins except Kinase.

5) Accuracy of QvR Blast vs All-versus-All-Blast:

To compare the accuracy of NC scores from both Blast methods, we sampled 1000 points from a dataset and computed the regression line from this data as illustrated in Figure 5. Almost 80% of the data (remaining data had QvR or AvA NC score < 0.3) is following the trend line shown in Figure 2 with strong correlation coefficient R^2 = 0.96. Therefore, we decided to compute Query versus Reference Blast due its computation efficiency and meaningfulness.

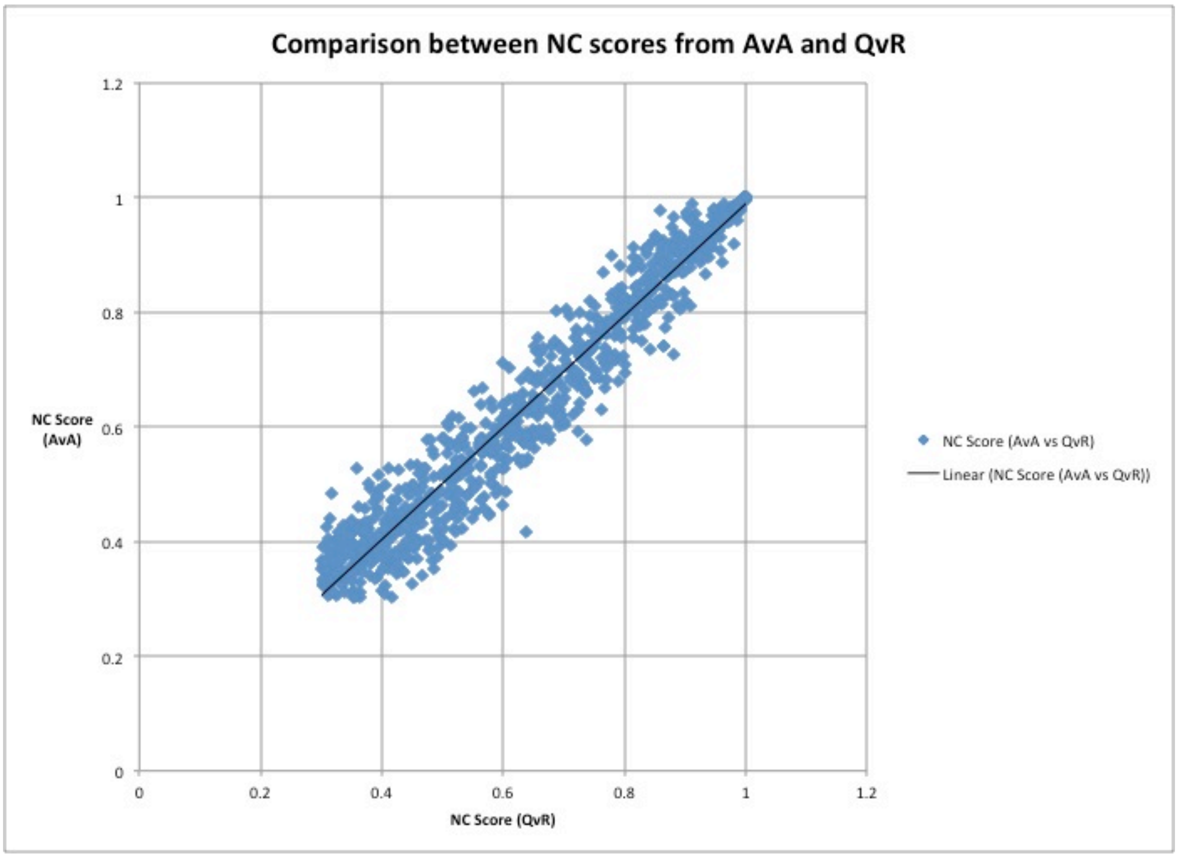

Figure 2 - Scatterplot of NC computed from all-versus all blast and Query versus Reference blast (our approach). This scatter plot demonstrates strong correlation of NC Scores between the two methods. Query Versus Reference Blast models the All versus All blast with a strong correlation coefficient $R^2 = 0.96$.

6) Interesting notes:

i) Why NC not BLAST for defining similarity

There are many criteria available for judging gene homology based on similarity but most of the recent and accurate ones take advantage of the network properties derived from Blast hits. HiFiX[17], for example, takes advantage of the profile HMMs that capture the average similarity in a gene family and based on this decides to merge or not merge two separate clusters in each run. The Neighborhood Correlation algorithm [16] by Song et al. belongs to the same class of algorithms that take advantage of the network properties based on Blast results. It bases its correlation scores on the shared versus unique neighborhood of each pair of genes in the graph. However, as opposed to other software of this category, it gives its answer in terms of a single standardized number between 0 and 1, which makes it easy to apply a threshold [31]. Furthermore, the accuracy of the Neighborhood Correlation algorithm has been shown to be much better than any of the algorithms that are based on applying any threshold on alignment length, E-values or bit scores of Blast [31]. Because of these properties, Neighborhood Correlation (NC) was chosen as an indicator of similarity between two genes. Furthermore, NC score, instead of Blast scores, have been used for calculating synteny scores, where it has given a further advantage of bounded and standardized scores as well.

ii) Advantages of using NC scores for calculating synteny scores

Algorithms that calculate synteny have generally used BLAST scores as the similarity measure required for calculating synteny scores. To the best of our knowledge, no

one has used any other measure for checking similarity in the vicinity of query genes. GenFamClust is the first algorithm that relies on the similarity correlation scores in the syntenic regions close to the query genes. The benefits of such an approach are similar to those of Neighborhood Correlation over raw bit scores. Unlike bit scores, the range of synteny score for any two genes is always in a definite range with zero implying lower limit and upper limit is fixed but method and neighborhood size dependent. Also, unlike syntenic scores using bit scores, the synteny score for GenFamClust is standardized. Thereby strength of synteny between two genes is directly shown as a relative number, on which a threshold can be applied. Moreover, the similarity score is based on evidence from a number of related genes and genomes, and not only on the direct similarity between neighboring genes.
